# Qualitative evaluation of a diabetes electronic decision support tool: views of users

**DOI:** 10.1186/1472-6947-12-61

**Published:** 2012-07-03

**Authors:** Qing Wan, Meredith Makeham, Nicholas A Zwar, Susanna Petche

**Affiliations:** 1 School of Public Health and Community Medicine, University of New South Wales, Sydney, NSW, 2052, Australia

## Abstract

**Background:**

Quality care of type 2 diabetes is complex and requires systematic use of clinical data to monitor care processes and outcomes. An electronic decision support (EDS) tool for the management of type 2 diabetes in primary care was developed by the Australian Pharmaceutical Alliance. The aim of this qualitative study was to evaluate the uptake and use of the EDS tool as well as to describe the impact of the EDS tool on the primary care consultation for diabetes from the perspectives of general practitioners and practice nurses.

**Methods:**

This was a qualitative study of telephone interviews. General Practitioners and Practice Nurses from four Divisions of General Practice who had used the EDS tool for a minimum of six weeks were invited to participate. Semi-structured interviews were conducted and the interview transcripts were coded and thematically analysed using NVivo 8 software.

**Results:**

In total 15 General Practitioners and 2 Practice Nurses completed the interviews. The most commonly used feature of the EDS tool was the summary side bar; its major function was to provide an overview of clinical information and a prompt or reminder to diabetes care. It also assisted communication and served an educational role as a visual aide in the consultation. Some participants thought the tool resulted in longer consultations. There were a range of barriers to use related to the design and functionality of the tool and to the primary care context.

**Conclusions:**

The EDS tool shows promise as a way of summarising information about patients’ diabetes state, reminder of required diabetes care and an aide to patient education.

## Background

General Practitioners are playing an increasing role in delivering effective care to prevent the progression of type 2 diabetes. There is evidence that systematic primary care can achieve standards of care as good or better than hospital outpatient care for type 2 diabetes [1]. However, diabetes is a complex disease to manage and there are many barriers to patients achieving their optimum treatment and management goals. For example the AusDiab study has shown that only one in seven patients being treated for type 2 diabetes is achieving the three key targets of glucose control, blood pressure management and cholesterol levels [2].

The Pharmaceutical Alliance (a collaboration between Elli Lilly Australia Ltd, Merck Sharpe and Dohme Australia Ltd and Sanofi Aventis Australia Ltd), in consultation with an expert reference group commissioned the development of an electronic decision support (EDS) software tool designed to support primary care practitioners in the care and management of their patients with type 2 diabetes. The tool is based on Diabetes Australia/ RACGP guidelines [3] for the management of type 2 diabetes and was designed to link and share data with the General Practitioner's (GP) clinical records systems. The software tool links with two of the leading desktop software products: Best Practice™ and Locum™. Key features of the tool are described in Table 1.

**Table 1:** Features of the Diabetes EDS tool


**Key features**	·a diabetes ‘toolbar’ showing the patient’s latest key measurements, highlighting whether these are at the recommended level or due for a check-up
	·brings critical information together in an easy-to-review format to cover all aspects of the patient’s condition
	·allows gps to proactively monitor the patient’s health status and progress to clinical treatment goals
	·links with existing clinical record systems enabling the data to be read from and recorded back into the core gp system
**Additional features**	·prompts informed discussion through interactive and informative summary screens
	·easy-to-use analytical screens and graphs to highlight the patient’s progress
	·linked to nhmrc and da / racgp diabetes management guidelines
	·provides a workflow oriented approach to data entry
	·chronological and cumulative summary
	·task reminders
	·access to resources for gps and patients
	·direct links to useful patient information and support tools
**Further information**	·further information and example of screen grab available at http://www.consultdave.com.au/index.html (accessed 28 may 2012).

The aim of this qualitative study was to evaluate the uptake, use and perceived value of the EDS tool in the care of patients with type 2 diabetes and describe the impact of the EDS tool on the primary care consultation from the perspectives of general practitioners and practice nurses.

## Methods

### Design

This was a qualitative study of semi-structured telephone interviews with users of the EDS tool.

### Ethical Approval

Ethics approval was given by the University of New South Wales Human Research Ethics Committee (approval number HREC 09064). The research was conducted in accordance with the Helsinki Declaration.

### Recruitment

There were 68 General Practitioners (GPs) and 9 Practice Nurses (PNs) from nine Divisions in New South Wales, Western Australia and Victoria who were users of Best Practice or Locum software and who expressed interest in being part of a demonstration project to use and evaluate this tool. These GPs and PNs were invited to participate in the study by three rounds of letters, and followed by reminder phone calls and faxes to non responders. GPs and PNs who had used the EDS tool for at least six weeks were eligible to participate as it was considered that this was a reasonable amount of time to have had an opportunity to use the tool and form a view about its value. Eligible GPs and PNs who agreed to participate provided written informed consent.

### Intervention introduction into practices of the EDS tool

The EDS tool was installed in participating practices by a team from the Pharmaceutical Alliance. The GPs and PNs in the practices were encouraged to make use of the tool as they saw fit with no restriction on how often or with which type 2 diabetes patients it should be utilised. There was a variable degree of orientation provided to GPs and PNs, however, continuous technical support was offered by the Pharmaceutical Alliance team.

### Data collection

Data collection was completed independently by the team from University of New South Wales. GPs provided background information about themselves and their practice demographics by completing a questionnaire at the beginning of the study. After at least six weeks of using the EDS tool, GPs and PNs were interviewed by phone for approximately 20–30 minutes. Interviews were audiotaped, and professionally transcribed and checked. The interviews explored: use of the EDS tool; impact of the tool on the consultation process; impact of the tool on diabetes care; barriers to the use of the tool and suggestions on its improvement.

### Data analysis

The qualitative data from GP, PN and patient interviews were coded and analyzed using NVivo 8 software [4]. The accuracy of transcripts was checked by QW prior to being transferred to the QSR NVivo 8 software for analysis. Out of 17 interviews, four interviews (three GPs, one PN) were pilot coded by QW, MM, SP and NZ together to develop an initial coding framework. Where there were differences these were discussed to resolve them until consensus about the coding was reached. Based on the coding framework, QW coded the rest of the interviews and modified the nodes in the analysis process to best fit the data. Thematic analysis was used for qualitative data analysis [5,6].

## Results

### Recruitment and GP background findings

Out of 68 GPs invited: 22 consented to participate in the study; 14 refused and 32 did not respond to the invitation. The practices of the 46 GPs who either refused or did not respond were contacted to try to ascertain why they were not interested. The reasons given were: the GP did not use the EDS tool; were too busy; had no interest, moved to other practices or unknown. Out of nine PNs invited, all responded with two consenting to participate in the study and seven nurses declining to take part. The reasons for not participating were: not using the EDS tool or unknown reason.

In total, 22 GPs and two 2 PNs (who were involved in diabetes care and education) consented to participate in this study. All 22 GPs completed the background information questionnaire (Table 2) and 15 of them plus 2 PNs took part in the semi-structured interviews. Semi-structured interviews were also completed with two practice nurses.

**Table 2:** GPs’ demographic and background information

**GPs**	**Frequency**
Total	22
Gender	Female
Male	8
14	Age groups
<45	45–54
>54	10
5	7
Practice size	Solo
2–5	>5
0	5
17	Location
Rural	Urban
2	20
Have access to diabetes guidelines when consulting	No
Infrequently	Frequently
3	15
4	Using electronic records when consulting (no paper records )
22	

### Interview findings

Thematic analysis resulted in 3 major themes which were described as below.

### Use of the tool and impact on diabetes care

When GPs and PNs were asked about their reasons for using the EDS tool, many reported that it was useful in giving them a quick summary of their patients’ type 2 diabetes care; the tool also provided a good reminder of their patients’ risk factor information and any related care that was outstanding. This was the most common reason for their use of the tool.

"*It gives us a … very quick summary and … very good reminder of what needs to be done. GP1009*"

"*Highlighted whether it’s been done recently, more to the point whether it hasn’t been done recently. PN 0201*"

The design of the EDS tool offers multiple screens which involve different functions supporting the care of patients with diabetes. These multiple functions were tailored by participating GPs and PNs and used to a greater or lesser degree in managing different individual patients. Many GPs reported that the most useful function was the side bar screen which provided an overview at a glance of all the important factors in diabetes management and a reminder of the outstanding items in the patient’s type 2 diabetes care. Overall, those two functions were viewed as the tool’s key role in diabetes care and we the most commonly used features.

"*If you don’t have a prompting tool you may forget something and so on. Here it’s quite hard to forget something because it’s flashing in red there so it almost makes you want to do those things in a way. -GP0101*"

"*Acts as a reminder to check certain things like weight, blood pressure, smoking and so on regularly and it also reminds me to do blood sort of on a regular basis. It’s more like a reminder system for me to be honest but I know anyway but it just keeps me, reminds me to do again. -GP1303*"

The extent of use of some of the more detailed aspects of the tool in practice is variable, partly because of the tool’s shortcoming such as the speed of the software causing some delays in the consultation when users attempt to access these sections, and partly because of a lack of users’ understanding of the full set of features contained within the tool.

"*The more you use it the more your realise it is useful -* GP1303"

Both GPs and PNs reported using the tool as a resource to assist in the process of education of patients about diabetes and the targets for control of glycaemia and macrovascular risk factors. The fact that information was presented in a graphical format and presented on the screen was considered a visual aide to patient understanding.

"*More understanding on the basis of the patient. If you just talk and you don’t illustrate, very little of it gets retained, whereas illustrations help the patient to link the information to something else in their brain and they’re more likely to remember it. –GP1701*"

One of the PNs not only used the tool as a visual aide but also used it to provide print out patient educational material relevant to the discussion.

"*It’s nice to give a visual thing for the actual patient themselves. It makes them feel included when you share that information with them and because it comes from their file also they think that it makes it seem a little bit more personal. Print it out into, onto the particular specified screen that you like. So say for example they’re not to increase their activity then you can expand and go into that and then you can print out information so you can take it step by step –PN 0201*"

Most GPs and both PNs felt that the use of the tool positively affected their management of patient lifestyle risk factors with the focus determined by individual need. This is because they were reminded or prompted by the tool to do so.

"*I discuss it more often because I’m reminded about it because of the software. –GP1702*"

The tool reminded GP to reinforce to their patients the nature of diabetes lifestyle management which needed to be addressed.

"*We’re always reminded about things like their weight and their exercise and things like that and whether they smoke or drink or all those other things that go on that screen so it’s probably just reinforcing it again. –GP1706*"

The GPs and PNs saw less value and reported less use of the tools of functions such decision support on use medicines, making referrals or providing follow-up reminders. This was often as the clinicians did not see a need for decision support in this aspect of care. For example in the area of medicines for diabetes one GP commented:

"*I think it doesn’t affect someone if your knowledge of diabetes is substantial but if your diabetes knowledge is not that good it offers a lot of advice of what might be the best thing to do with –it does depend on the doctor’s level of knowledge. –GP0201*"

The EDS tool provides diabetes guideline information for clinicians to access during the consultation. Many GPs and both PNs reported that they were already familiar with the guidelines for diabetes management, but that the tool did reinforce application of the guidelines in daily work.

"*I think the guidelines are essentially buried into the tool so it’s what tells me whether or not the targets are reached so it’s, it’s almost like a, a reminder in the background about the guidelines so the tool applies to guidelines and informs me how I’m going with each particular patient. –GP0201*"

There was a range of views on the value of the EDS tool in completing an annual diabetes cycle of care. A diabetes cycle of care is a defined list of care processes to be conducted each year and the completion of the cycle attracts an incentive payment to the GP from Medicare Australia. While only a small number of GPs perceived any value of the tool in this regard both PNs thought the tool helped in getting the cycle of care completed.

"*A prompt for the GPs to remind them the patient is a diabetic, to send them to me for their annual cycle of care if it’s due. PN0401*"

### Impact on the consultation process

The perceptions of GPs varied on the impact of the tool on consultation times. Many GPs felt that they tended to spend longer with their patients when using the tool compared their usual consultations, however this was because they were using the tool to provide better quality of care for their patients. One of the PNs interviewed felt that the consultation was lengthened, and the other reported no change.

Problems with the functionality of the tool software were perceived by some GPs as costing time. Some GPs thought the EDS tool software itself slowed down their IT systems and did not always import information appropriately which resulted in the need of adding information manually. Additionally, some felt the tool was distracting them at times from the purpose of their consultation, in particular when the patient was not attending for a problem related to their diabetes care.

"*I mean even to load it up and stuff just takes too much time and it slows down the consultation quite a lot you know. –GP1009*"

"*I think it takes a bit longer because it’s you know a lot of ticking and putting things in – it doesn’t always automatically do it for some reason. GP1303*"

The use of the tool as a visual aide to patient education has been previously discussed. The majority of GPs said they shared the information displayed by the tool to discuss issues such as pathology results, cardiovascular risk factors, guideline recommendations and metabolic targets. It also made it easier to illustrate to patients what they were talking about. The majority of the GPs and one of the PNs thought their communication with patients improved by using the EDS tool. This was because that the tool provided detailed information to them when needed and removed the need to spend time searching different fields of their desktop software.

By sharing the screen with the patients, many GP believed that their patients’ understanding of their diabetes care improved.

"*If you just talk and you don’t illustrate, very little of it gets retained, whereas illustrations help the patient to link the information to something else in their brain and they’re more likely to remember it. –GP1701*"

Related to improved communication more than half of the GPs thought use of the tool was of benefit to the doctor-patient relationship.

"*I think doing it correctly would reinforce the doctor/patient relationship and also create more rapport. –GP1401*"

One of the PNs thought the tool also helped her communicate with the GPs in the practice about the care provided.

"*Because when you’re printing out it will document that down so you can – instead of writing everything down you, you can communicate more effectively to the GP. –PN0201*"

However a few GPs thought the tool interfered with communication with the patient.

"*It [the tool] sort of diverts my attention…… It makes me focus more on the computer. -GP1705*"

Some GPs expressed a perception of increased patient satisfaction and engagement with their diabetes care in the consultation as a result of seeing their doctor’s interest in using the EDS software.

"*If I’m focussing on the screen often it will make the patient focus on thescreen as well and then I’d, I would point a few things out and then show, show a few things on the screen. –GP0101*"

"*The patient gets happy about it because the thing is they know the doctor is taking a particular interest in them, so I think that’s useful, yes. -GP 1007*"

Most importantly, no GPs or PNs reported that their patients had any negative experience associated with the use of the tool during consultations.

### **Problems/barriers to use and suggestions for improvement**

There were a range of problems identified with the tool and barriers to use. These included problems with the functionality of the tool itself and barriers related to the skills of the users. These problems and barriers are summarised in Table 3.

**Table 3:** Barriers to use of the tool

**Barriers related to the tool**	**Barriers related to users**
·Loading or opening speed too slow	·Users’ poor knowledge with the tool’s functions
·Sometimes pathology results not fully uploaded or no results in the tool*e.g. eGFR, cholesterol or urine albumin*	*e.g. Not aware of other functions except for the side bar*
·Some information not included in the tool	·Time pressure
e.g. *information on vaccinations, cardiovascular risk*	·No financial incentive to GPs to use the tool
·Problems with the tool’s screen*e.g. Screen interferes with the existing window, too big and cannot be minimised*	·Not fitting the use of the tool with the consultation process
·Lengthened the consultation time	
·Cannot add in new progress notes	
*e.g. Foot check notes, eye check notes, action and improvement notes*	
·Wrong alert of results*e.g. sometimes alerts everything in red (as abnormal), still no change even when changing the values manually*	
·No summary information report for diabetes cycle of care	

There was a range of suggestions for improvements. These included addressing problems with functionality such as the software running slowly, improving the printing function and also improving integration of the tool with the practice clinical software program. There were also suggestions for new functionality such as providing automatically updated diabetes guidelines via a pop-up or link and being able to modify the goals of treatment for individual patients.

A common suggestion for improvement was a request for a summary information report for the diabetes annual cycle of care. Some GPs were keen to see if the tool could technically be configured to better assist them in determining when they had completed a diabetes annual cycle of care and somehow trigger them to automatically bill this item.

"*You would want to know when the last cycle was completed and whether you have been able to satisfy all the criteria for a cycle of care for example, and if the tool could produce a summary of the cycle of care for example. –GP0101*"

There was variability into the extent of training and support that the GPs and PNs had received. Nearly all participants thought that introductory training was necessary and there were a range of suggestions on this and ongoing support

"*I think it does require a fairly good introduction, introductory session to show where the effectiveness of the tool can, can come in, where you can benefit from this and how to fit it into a consultation. –GP0101*"

Suggestion on the content for the introductory training included demonstrating the range of capabilities of the tool, practising on dummy patients and testing on the practice’s own software system.

In contrast to the introductory training, only some participants supported the need for providing continuous support as they felt that the tool was pretty straight forward.

"*I think because I mean it works actually quite well on its own. I don’t think there’s much training necessary really. Maybe you know initial training and that’s it. I don’t think ongoing support is necessary really. –GP1303*"

Among those who thought ongoing support was needed, suggestions for providing this included having regular follow-ups by practice visits or telephone calls and/or a telephone helpline.

## Discussion

Health providers are keen to have a tool that helps support the care of their diabetic patients in their generally limited consultation times. There is evidence that integrated electronic decision support tools can be effective in changing routine behaviours amongst clinicians, as well as improving quality of care provided, but the extent of impact on patient clinical outcomes over time remains unclear [7-10]. The EDS tool has a number of features that have been found in a systematic review to be critical to success of clinical decision support systems. These are automatic provision of decision support as part of clinician workflow; provision of recommendations rather than just assessments; provision of decision support at the time and location of decision making; and computer–based [11].

In this study, the most common reason for using the tool was its role in providing a summary of the current state of the patients’ diabetes and reminder of required diabetes care. These features were also the most useful functions of the tool experienced by users. GPs and PNs used the tool in a range of ways to involve patients and provide better quality care for type 2 diabetes: sharing screens with patients, increased discussion and better communication with their patients. To some extent the tool also reinforced GPs and PNs use of current diabetes guidelines. Even though the use of the tool was likely to lengthen the consultation time, the GPs and PNs thought it helped improve patient engagement and quality of care.

In terms of its specific impact on the provision of care for diabetes, it was found that most GPs and both PNs used the tool mainly as an aid in managing patient lifestyle changes rather than for other purposes such as helping manage medications prescribing, referral and follow-up. Therefore the full capacity of the tool was not being used by most study participants. Generally, the tool did not help GPs or PNs in managing the annual cycle of care recommended by diabetes guidelines, probably as it did not directly summarise or link with this cycle of care.

In this study, a number of barriers were found to the use of the tool and suggestions were provided in interviews by GPs and PNs on improving the tool: both relating to the tool’s shortcomings and training and support to users. Some of those findings are consistent with other research, which identified that barriers to the uptake of electronic decision support tools included a lack of a business case, shifting of costs for data collection and management to the clinician, uncertainty about the optimal level of decision support, lack of technical and semantic standards, and resistance to electronic decision support tool use by the time conscious GP [12].

This study has some limitations. Participating GPs, PNs and patients are willing participants who showed interest in this study. This sampling strategy would affect to some extent generalizing the study findings beyond those participants. This qualitative study is only a short-term study. It cannot tell us if those positive impacts of the tool on consultation process and delivering care to diabetic patients would maintain in a longer time and how effective the tool would be in improving patent clinical outcomes. The study provided qualitative information only and further evaluation is needed in the form of quantitative measurements of change in GP and PN behaviour and in patient level measures of process and outcomes of care related to use of the decision support tool. Ideally this would be in a randomised controlled trial as though there is evidence that an electronic decision support system can improve process of care and some clinical markers of quality of diabetes care the published literature is limited [13].

An issue for all information technology tools is whether the uptake and use will be maintained and how a particular tool will fare in the face of competition. This evaluation does not answer these questions but they are relevant ones in the Australian context where new electronic decision support tools are being disseminated such as the PrimaryCare Sidebar which is supported by the Royal Australian College of General Practitioners [14].

## Conclusions

Overall this qualitative analysis of GP and PN interviews found that the EDS tool had a positive impact on the quality of care of type 2 diabetes, and improved the ability of GPs and PNs to more effectively manage consultations in patients with type 2 diabetes, and the EDS tool was both feasible and practical for use in the clinical setting.

Findings from this study also provide the basis for future modification of the EDS tool and furthering its ability to fit into the daily practice of managing patients with type 2 diabetes. Further work on the tool could address implementation strategies of improving the uptake of the tool, with more structured training, enhancing certain functions such as links to the annual cycle of care and its speed when integrated into patient management software.

## Competing interests

The authors declare they have no competing interests

## Authors' contributions

All authors contributed to the conception and design of the study and to interpretation of the data. QW, NZ, MM and SP all contributed to the development of the interviews and the coding framework. QW conducted the interviews, conducted the analysis and completed the initial report. NZ drafted the paper. All authors read and approved the final manuscript.

## Pre-publication history

The pre-publication history for this paper can be accessed here:

http://www.biomedcentral.com/1472-6947/12/61/prepub
